# miR398-SlCSD1 module participates in the SA-H_2_O_2_ amplifying feedback loop in *Solanum lycopersicum*

**DOI:** 10.1016/j.jare.2025.04.035

**Published:** 2025-04-22

**Authors:** Xiujuan Wang, Xinshan Zhang, Yuanyuan Liu, Lei Ru, Guochao Yan, Yunmin Xu, Youjian Yu, Zhujun Zhu, Yong He

**Affiliations:** a College of Horticulture Science, Zhejiang A&F University, Hangzhou 311300 Zhejiang, China; b Key Laboratory of Quality and Safety Control for Subtropical Fruit and Vegetable of Ministry of Agriculture and Rural Affairs, Hangzhou 311300 Zhejiang, China

**Keywords:** Salicylic acid, Reactive oxygen species, miR398, Tomato, H_2_O_2_

## Abstract

**Introduction:**

Salicylic acid (SA) is essential for immune response signal transduction in higher plants, with its signaling thought to be enhanced through interactions with reactive oxygen species (ROS). However, the exact mechanisms behind this SA self-amplifying signaling are still not well understood.

**Objectives:**

In this study, we report the involvement of the miR398b-SlCSD1 module in the SA-H_2_O_2_ amplifying feedback loop in tomato (*Solanum lycopersicum*).

**Methods:**

Experiments were conducted using various concentrations of SA to assess its impact on ROS metabolism and the expression of *SlCSD1* and sly-miR398. CRISPR/Cas9 was employed to knock out sly-miR398 and *SlCSD1*. Bioinformatics analyses, dual-luciferase reporter assays (Dual-Luc), and electrophoretic mobility shift assays (EMSA) were used to identify SA-responsive transcription factors and validate their regulation of sly-miR398b. The role of miR398 in endogenous SA synthesis was examined using overexpression and knockout tomato lines.

**Results:**

Low SA concentrations stimulated H_2_O_2_ accumulation, increased superoxide dismutase (SOD) activity, and suppressed *sly-miR398* expression, effects absent in *NahG* plants with reduced SA levels. Knockout of SlCSD1 via CRISPR/Cas9 partially inhibited SA-induced H_2_O_2_ accumulation, confirming *SlCSD1*′s role in SA-dependent H_2_O_2_ signaling. Furthermore, Dual-Luc and EMSA results revealed that TGACG-sequence-specific binding protein 2 (TGA2) mediated the regulation of miR398-SlCSD1 module by SA in tomato. Additionally, overexpression and mutation of sly-miR398b promoted SA synthesis via the phenylalanine ammonia-lyase (PAL) and isochorismate synthase (ICS) pathways, highlighting its regulatory role in SA biosynthesis.

**Conclusion:**

Taken together, our results shed light on the involvement of the miR398-SlCSD1 module in the SA-H_2_O_2_ amplifying feedback loop, providing new insights into SA signaling in tomato. These findings contribute to understanding SA-ROS interactions and offer a potential strategy for enhancing stress tolerance in crops by targeting microRNA-regulated pathways.

## Highlights

• miR398-CSD1 is involved in the H_2_O_2_ signaling by SA.
• TGA2 mediated the inhibition of SA on miR398-CSD1 module.
• Fluctuations in miR398 levels induce SA synthesis.

## Graphical abstract

**Figure:**
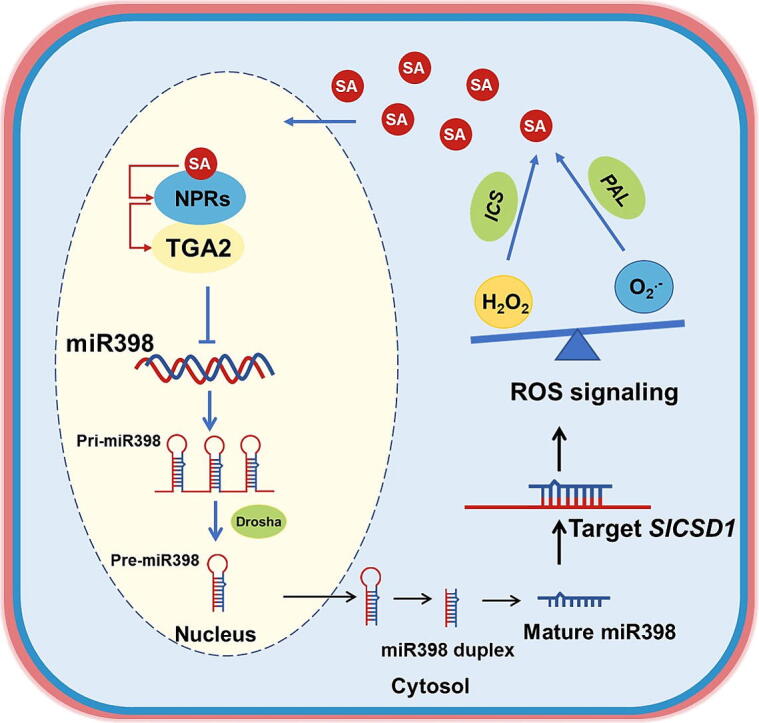

## Introduction

Salicylic acid (SA) is a plant defense hormone that plays a crucial role in defending against abiotic and biotic stresses [1]. When plants are attacked by pathogens, SA is produced and initiates local pattern-triggered immunity (PTI) and effector-triggered immune responses (ETI), with the latter resulting in programmed cell death and the induction of systemic acquired resistance (SAR) [2]. Under abiotic stress conditions, SA promotes H_2_O_2_ production, which aids in stomatal closure to conserve water during drought and enhances antioxidant enzyme activity to mitigate oxidative stress caused by salinity or low temperatures [[3], [4], [5]], Supplemental Fig. S1. The SA signaling is amplified through interactions with reactive oxygen species (ROS), applications of H_2_O_2_ and SA to plants induced each other, suggesting their involvement in a self-amplifying feedback loop [6,7]. It has long been recognized that SA induces the production of H_2_O_2_ through the increased activity of Cu/Zn-superoxide dismutase (CSD), which converts superoxide into H_2_O_2_, and the decreased activity of catalase (CAT) and ascorbate catalase (APX) as well [8,9]. Moreover, the expression of *CSD* is up-regulated by exogenous SA [10]. In general, SA activates gene expression through promoter *cis*-elements, such as the TCA element (TCAAGA) and the W box (TTGACC) [11]. However, no SA-responsive *cis*-element was identified in the promoter region of *SlCSD1* in *Solanum lycopersicum* (Supplemental Table S2), indicating the presence of post-transcriptional regulation of *SlCSD1*.

Small RNAs regulate post-transcriptional gene silencing by influencing mRNA stability or translation [12,13]. A conserved miRNA, known as miR398, cleavages the mRNA of *CSD* in both *Arabidopsis thaliana* and tomato [14]. Similar to other plant miRNAs, primary miR398 (pri-miR398) is transcribed by RNA polymerase II from *MIR398* genes and then processed into 21-nt miRNA duplex by proteins like HYPONASTIC LEAVES1 (HYL1), DICER-LIKE1 (DCL1), and SERRATE (SE) [15]. The duplex is then exported to the cytoplasm, and loaded onto the RNA-induced silencing complex (RISC), where it targets *CSD* transcripts [16]. miR398 is a well-characterized stress-responsive microRNA, implicated in a broad range of abiotic and biotic stress responses, including salinity, high temperature, copper and iron concentrations, as well as bacterial, fungal, or viral infections [[17], [18], [19]]. Overexpression of sly-miR398 has been reported to increase superoxide levels by reducing SOD activity in tomato [20,21].

SA induces transcription by activating TGACG SEQUENCE-SPECIFIC BINDING PROTEIN (TGA) transcription factors [22]. Unlike other plant hormones, SA is recognized by two distinct classes of receptors: NPR1 and NPR3/NPR4, which play contrasting roles in the regulation of defense gene expression [23]. NPR1 functions as a transcriptional activator, while NPR3/NPR4 acts as transcriptional repressors. When SA levels reach a certain threshold, it promotes the monomerization of NPR1 and inhibits the activity of NPR3/NPR4. Both NPR1 and NPR3/NPR4 interact with transcription factors [24,25]. The *Arabidopsis* genome contains 10 TGA genes, which are grouped into five clades based on their phylogenetic relationships. Clade II members (TGA2, TGA5, and TGA6) have been extensively studied and their central role in defense responses is well documented [26,27]. Moreover, Clade II TGAs are considered to be involved in the induced expression of defense genes by SA in *Arabidopsis* [[28], [29], [30]].

The SA-ROS amplifying feedback loop has been widely recognized, with the induction of *CSD* by SA considered a critical initial step. However, the precise mechanism through which SA regulates *CSD* remains unclear. We hypothesize that the miR398-SlCSD module, along with TGA2 transcription factors, serves as a mediator linking SA to ROS production. To verify this hypothesis, we investigated the effects of SA on ROS content, SOD activity, and the expression of miR398 and *SlCSD1* using exogenous SA treatment or *NahG* transgenic plants with reduced contents of active SA. Furthermore, we identified TGA genes in the tomato genome and examined their interaction with the miR398 promoter. We also constructed *csd1* mutants to test the dependence of SA regulation on ROS signaling. Moreover, we examined the effects of miR398 levels on SA synthesis and signaling. Our results provide a comprehensive understanding of the exact mechanisms underlying SA self-amplifying signaling.

## Materials and Methods

### Plant materials, growth conditions and SA treatment

The seeds of *Solanum lycopersicum* 'Micro-Tom' were germinated in the dark at 30 °C for 2 days, and then grown in a commercial substrate (“Gold No. 3″, Hangzhou Jinhai Agricultural Technology Co. LTD, China) under controlled conditions for approximately 28 days. Afterward, the primed seedlings with 4 true leaves were transplanted into a hydroponic system [20] and cultured in 10-liter plastic containers containing aerated full-strength Japanese Garden Formula [31]. The plants were kept in normal greenhouse condition with a day/night temperature of 28 °C/20 °C, a light intensity of 800 μmol m^−2^ s^−1^ and 14-hour photoperiod, and 70 % relative humidity. To study the effect of SA, a series of concentrations (0.01 mM, 0.1 mM, 0.5 mM, and 1 mM) were added to the nutrient solution and maintained for 7 days. The plants without SA in the solution served as the control, with four trays (16 plants in total) included in each treatment and the control.

### Generation of transgenic plants

The overexpression and mutation of sly-miR398b plants were with background of “Mirco-Tom”. The T3 generation of overexpressed tomato was obtained from our lab [20]. To generate sly-miR398b loss-of-function mutants, CRISPR/Cas9 technology was used following established protocols [32]. Single-guide RNAs (sgRNAs) targeting the sense strand of the miR398b gene were designed using an online tool (https://www.biogle.cn), which includes features for specificity and off-target prediction. The sgRNA sequences (Supplemental Table S1) were synthesized and annealed by mixing 1 μL each of forward and reverse oligonucleotides (10 μM) with 18 μL annealing buffer. This mixture was incubated at 95 °C for 3 min and then cooled to 20 °C at a rate of 0.2 °C/s. The resulting double-stranded sgRNAs were cloned into the PYLCRISPR/Cas9pUbi-N binary vector, which utilizes the 35S promoter to drive Cas9 expression. Integration of sgRNAs into the CRISPR/Cas9 vector was performed by mixing 1 μL of double-stranded sgRNAs, 1 μL of enzyme mix, 2 μL of the CRISPR/Cas9 vector, and 6 μL of water, followed by incubation at 20 °C for 1 h. The vectors were then transformed into *Agrobacterium tumefaciens* (GV3101) for infection of cotyledon callus. Transgenic plants were selected on Murashige and Skoog (MS) medium containing 25 μg/mL hygromycin B. From this process, nine T_0_ transgenic lines of sly-miR398b mutants were generated, of which seven were confirmed to be homozygously mutated. PCR screening validated homozygosity, and stable T_3_ lines were obtained for further experiments.

To reduced active SA content in tomato, we got the *NahG* overexpression transgenic tomato plants with background of “Money-Maker” from Professor Yichen Lu at Nanjing University of Technology.

To investigate the dependence of SA on *SlCSD1, SlCSD1* was mutated using CRISPR/Cas9 technology with the ‘Ailsa Craig’ tomato variety as the background. sgRNAs targeting *SlCSD1* (Supplemental Table S1) were designed using an online tool (https://www.biogle.cn). The sgRNAs were cloned into the PYLCRISPR/Cas9pUbi-N vector and subsequently transformed into *Agrobacterium tumefaciens* strain GV3101. Seven primary *SlCSD1* transgenic lines (T_0_) were generated, five produced successful mutations, one of which produced no mutations, and one showed off-target effects. PCR screening was performed to obtain homozygous stable trait T_2_ lines (*csd1-1*, *csd1-2*) for further experiments. The primer sequences used are listed in table S1.

### Determination and staining of H_2_O_2_ and O_2_^.-^

To measure the content of H_2_O_2_ and O_2_^.-^, approximately 0.3 g of tomato leaves were harvested. For the extraction of O_2_^.-^, the leaves were homogenized in 65 mM phosphate buffer solution (PBS, pH 7.8) and then centrifuged at 12,000 g for 15 min at 4 °C. The production rate of O_2_^.-^ was determined using the hydroxylamine oxidation method described by Elstner and Heupel [33]. For the extraction of H_2_O_2_, leaves were homogenized in 1.5 mL HClO_4_ (1 mM) with insoluble Polyvinyl pyrrolidone (PVP), followed by centrifugation at 12,000 g for 10 min at 4 °C. Take 60 μL of the supernatant, add 600 μL of eFOX reagent (250 μmol∙L^−1^ ammonium iron (III) sulfate, 100 μmol L^−1^ sorbitol, 100 μmol L^−1^ demethyl yellow, 1 % anhydrous ethanol, 25 mmol L^−1^H_2_SO_4_), measure the absorbance at 560 nm, and calculate the H_2_O_2_ concentration using the standard curve. The content of H_2_O_2_ was determined using the method of Cheeseman [34].

For the localization of the O_2_^.-^ and H_2_O_2_, leaves were stained with 0.1 % nitroblue tetrazolium (NBT) and 0.1 % 3,3-diaminobenzidine (DAB), respectively, according to the method described by Jabs et al. [35] and Vanacker et al. [36].

### SOD activity assay

SOD activity was determined by the method described previously [37]. Approximately 0.3 g of fresh tomato leaves were ground in 2.0 mL of lysis buffer (25 mM PBS pH 7.8, 0.2 mM EDTA, 2 % PVP). The mixture was then centrifuged at 12,000 g for 20 min at 4 °C. The supernatant was collected for SOD assay, which was calculated by its ability to inhibit the photochemical reduction of NBT.

### RNA isolation and transcript quantification

Total RNA was extracted from 0.3 g leaf using Trizol^TM^ reagent (Invitrogen, Thermo Fisher Scientific, USA). cDNA was synthesized from RNA using a Superscript first-strand synthesis kit (Takara, RR047A, Japan). Quantitative PCR was performed on an iCycler PCR machine (Jena, qTOWER3G, Germany) using TB Green PCR kits (Takara, Japan). The reference gene used for functional genes was *SlACTIN*, and *SlU6* was employed as a housekeeping gene for miR398 expression analysis. The primer sequences are detailed in Supplemental Table S1. Gene expression levels were calculated with 2^-△△Ct^ method [38], normalized to those of wild-type control plants.

### Dual-luciferase reporter assay

Experiment I: miR398b target gene validation. The *p35S::pri-miR398b* construct was generated by amplifying a 410 bp sequence of *MIR398b* gene from wild type of “Micro-Tom” genomic DNA using the primers listed in Supplemental Table S1. The PCR product was cloned into the *PGreen 62-SK* vector using LR Clonase II (Vazyme, China), which used for cloning and expression of constructs in transient assay. *PmirGLO-SlCSD1* was generated by cloning a 400 bp fragment (containing the target site of *SlCSD1* by miR398) into the *PmirGLO* vector using LR Clonase II (Vazyme, China).

Experiment II: the interaction of sly-miR398b with TGA1/TGA2. The *pmiR398b::LUC* construct was created by amplifying a 2000 bp segment of the MIR398b gene from genomic DNA of “Micro-Tom”, using the primers listed in supplemental Table S1. The resulting PCR fragments were then integrated into the *pGreenII 0800-LUC* vector via LR Clonase II (Invitrogen) recombination, which employed for luciferase reporter assays to evaluate promoter activity. The *MUT-pmiR398b::LUC* construct was generated by introducing a mutation into the *pmiR398b::LUC* construct, specifically changing the sequence from 5’-TGACG-3’ to 5’-ACTGC-3’ using site-directed mutagenesis method. The constructs *p35S::TGA1* and *p35S::TGA2* were generated by amplifying segments from wild type genomic DNA and cDNA, respectively, using the primers listed in Supplemental Table S1. The PCR products were subsequently integrated into the *PGreen 62-SK* vector using LR Clonase II (Vazyme, China) recombination.

The plasmids were introduced into Agrobacterium tumefaciens (strain GV3101) using the freeze–thaw method. After three-to-four-week-old of growth under standard conditions, *Nicotiana* leaves were infiltrated with the transformed *Agrobacterium* (OD_600_: 0.6). The infiltration buffer used contained 10 mM MgCl_2_, 10 mM MES, and 200 μM acetosyringone. Fluorescence signals were observed using a Charge-coupled Device (CCD) camera 2 d post-infiltration under dark conditions. Firefly and Renilla luciferase activities were quantified using a dual-luciferase assay kit (Yeasen Biotechnology) and measured with a Synergy two multi-mode microplate reader (BioTek Instrument, USA) [39].

### Electrophoretic mobility shift assay

For the prokaryotic expression assay, the full-length coding sequences of *SlTGA1* and *SlTGA2* were amplified and cloned into the pMAL vector (maltose binding protein, MBP) individually. MBP-tagged fusion proteins were expressed in Escherichia coli (Rosetta) and purified using amylose resin columns following the protocols described by Seo et al. [40] and Hellman et al. [41]. The DNA probes labeled with biotin for EMSA were detailed in Supplemental Table S1. EMSA was conducted using the LightShift Chemiluminescent EMSA Kit (Beyotime Biotechnology, China). The reaction mixture, comprising 2 μL of EMSA/Gel-Shift binding buffer, 2 μg of purified fusion proteins, and 1 μL of 50 fM biotin-labeled annealed oligonucleotides, was incubated for 20 min at 25 °C. The reaction mixture was then electrophoresed on a 4 % polyacrylamide gel containing 3 % glycerol, transferred to a positively charged nylon membrane, and UV cross-linked. Biotin signals were detected and quantified using a Pierce Chemiluminescence Kit (Beyotime Biotechnology, China).

### Measurement of endogenous SA content

The leaves were collected 25 d post-germination for the assessment of endogenous SA content in accordance with the protocol detailed by Sawyer and Kumar [42]. Leaf samples (∼0.5 g) were pulverized in liquid nitrogen and blended with 4 mL of 5 % trichloroacetic acid. The homogenates were extracted with 46 mL of 65.2 % (v/v) ether solution for 12 h. The aqueous phase was heated with 18.5 % HCl to achieve a final concentration of 3.2 %, using a water bath at 80 °C for 1 h. This extraction was repeated three times, and the combined aqueous phase was concentrated under vacuum using a rotary evaporator. The concentrated extract was dissolved in 10 mL of a buffer solution containing equal volumes of methanol and acetone (pH 3.2). To normalize the SA concentration, all results were calculated and expressed as micrograms of SA per gram fresh leaf, ensuring accurate comparison across samples.

For the analytical process, all samples were filtered through a 0.3 μm microporous filter and subsequently subjected to HPLC utilizing an Agilent 1100 HPLC system coupled with an ESI-TOF 6220 mass spectrometer (Agilent Technologies, Santa Clara, USA). The mobile phase consisted of a buffer solution containing methanol and acetone (pH 3.2), each in equal proportions, and was maintained at a flow rate of 1.0 mL/min. A sample volume of 20 μL was injected into the HPLC system for subsequent analysis.

### Statistical analyses

The data were analyzed using non-parametric Kruskal-Wallis one-way analysis of variance (ANOVA) and compared (significance level *p* < 0.05). Statistical analysis was performed using SPSS® statistical software (version 26; IBM® Corporation). The described results were confirmed in at least one other independent experiment.

## Results

### Sly-miR398 is involved in the ROS metabolism induced by SA

**Fig. 1:**
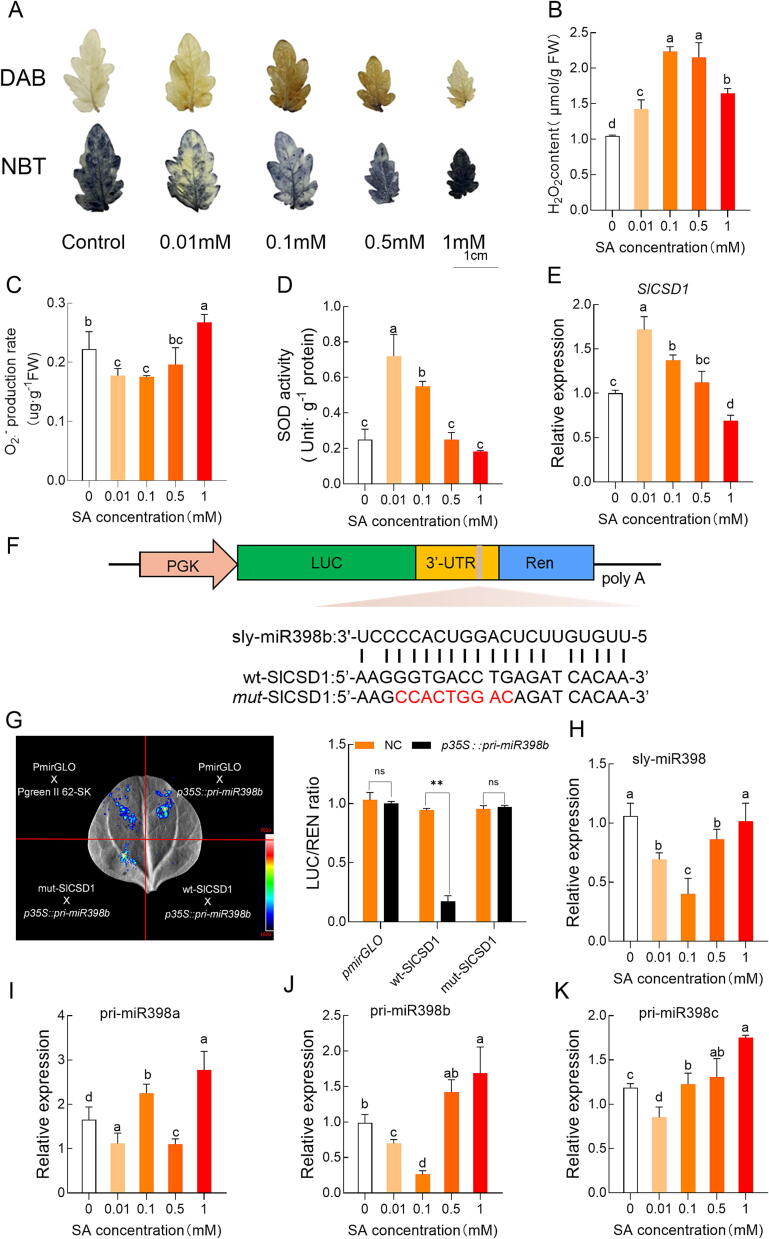
**SA induces the accumulation of H_2_O_2_, the activity of Superoxide Dismutase (SOD) and expression of *SlCSD1* at low concentrations, but it inhibits the expression of sly-miR398b.** (A-E) The effects of different SA concentrations on oxidative stress and antioxidant responses were analyzed in ‘Micro-Tom’ plants treated under normal conditions (control, 0 mM SA) and with SA (0.01 mM, 0.1 mM, 0.5 mM, and 1 mM) for 2 days. (A) Hydrogen Peroxide (H_2_O_2_) and Superoxide (O_2_^.-^) visualized using 3,3′-Diaminobenzidine (DAB) and Nitroblue Tetrazolium (NBT), (B) H_2_O_2_ content, (C) O_2_^.-^ production rate, (D) SOD activity, (E) *SICSD1* expression.(F–G) miR398 targeting experiment. (F) Schematic representation of miR398 targeting. Excess miR398 binds to target genes, triggering RNA instability and reducing LUC (firefly fluorescence). (G) The constructs containing wt-SlCSD1 or mut-SlCSD1 were separately expressed with p35S::pri-miR398b in *Nicotiana benthamiana* leaves. The ratio of Luciferase/Renilla fluorescence (LUC/REN) was measured. Data are presented as the means ± SD (n = 3) The asterisks indicate significant differences based on one-way ANOVA (*P < 0.05, **P < 0.01, ***P < 0.001, ns, no significance).(H-K) The effects of different SA concentrations on miR398 expression were analyzed in ‘Micro-Tom’ plants treated under normal conditions (control, 0 mM SA) and with SA (0.01 mM, 0.1 mM, 0.5 mM, and 1 mM) for 2 days. (H) miR398 expression, (I) pri-miR398a expression, (J) pri-miR398b expression, (K) pri-miR398c expression. Data are presented as the mean ± SD (n = 4). The statistical analysis of was based on one-way Analysis of Variance (ANOVA). Different letters indicate above the bars indicate significant differences (*P < 0.05*).).

The findings reveal a dose-dependent effect of SA on the levels of H_2_O_2_ and O_2_^.-^. Low concentrations of SA (0.01 mM and 0.1 mM) increased H_2_O_2_ content; whereas high concentrations of SA (1 mM) reduced it at 2-day time point of treatment. (Fig. 1A, 1B, Supplemental Fig. S2C). For instance, exposure to 0.1 mM of SA resulted in a 36.2 % increase in H_2_O_2_ content compared to control plants. Conversely, the accumulation of O_2_^.-^ showed an inverse trend, with low SA concentrations reducing O_2_^.-^ levels and higher concentrations (1 mM) enhancing its production (Fig. 1A, 1C). These observations demonstrated the contrasting effects of SA on the levels of H_2_O_2_ and O_2_^.-^.

The activity of SOD, which catalyses the conversion of O_2_^.-^ into H_2_O_2_, influences the levels of H_2_O_2_ and O_2_^.-^ in opposite directions. To examine the role of SOD in the changes of H_2_O_2_ and O_2_^.-^ brought about by SA, the activity of SOD and the expressions of related genes were assessed. The activity of SOD initially increased and then decreased as the concentration of SA increased (Fig. 1D). Similarly, the expressions of *SlCSD1* followed a similar trend as the activity of SOD when plants were treated with exogenous SA (Fig. 1E). In addition, the activation of H_2_O_2_ and SOD is also time-dependent (Supplemental Fig. S2A, B).

As previously stated, the promoter region of *SlCSD1* does not contain SA responsive elements, indicating that the changes in *SlCSD1* expression caused by SA might be due to post-transcriptional regulation. It is well documented that miR398 regulates *CSD1* in *Arabidopsis* through mRNA degradation [43]. To verify this, vectors of *wt-SlCSD1* and *mut-SlCSD1* (Fig. 1F) were generated and subsequently fused with *p35S::pri-miR398b*. The fluorescence intensity of *PmirGLO-SlCSD1* was weaker compared to *PGreen 62-SK*. However, when the target sequence of *SlCSD1* was mutated, the fluorescence intensity of *PmirGLO-SlCSD1-MUT* was restored. The ratio of firefly-luciferase (LUC) activity to Renilla-luciferase (REN) activity was reduced by miR398b mimics, but was restored in the *SlCSD1*-mutated vectors (Fig. 1G). These results suggest that *SlCSD1* was indeed targeted by sly-miR398b.

Additionally, the expressions of sly-miR398 when plants were subjected to various concentrations of SA. Low concentrations of SA (0.01 mM, 0.1 mM, and 0.5 mM) reduced the expression of sly-miR398, while high concentrations of SA (1 mM) did not result in significant changes (Fig. 1H). Moreover, the expressions of 3 members of the miR398 family (sly-miR398a located on chromosome 11, sly-miR398b located on chromosome 5, and sly-miR398c located on chromosome 12) in tomato were analyzed using real-time quantitative PCR (qRT-PCR). The expression pattern of pri-miR398b was more closely aligned with that of sly-miR398 compared to pri-miR398a and pri-miR398c (Fig. 1I–K). As a result, sly-miR398b was selected for further investigation.

Taken together, these results indicated that SA promoted the accumulation of H_2_O_2_ and sly-miR398 may play a pivotal role in this process.

### SA-degrading *NahG* plants up-regulated the expression of sly-miR398

To gain further insights into the impact of reduced SA on ROS metabolism and sly-miR398 expression, experiments were conducted experiments using *NahG* plants, which are characterized by overexpression of *NahG* and consequent reduction in active SA content, which was significantly smaller in *NahG* plants compared to wild-type plants.

**Fig. 2:**
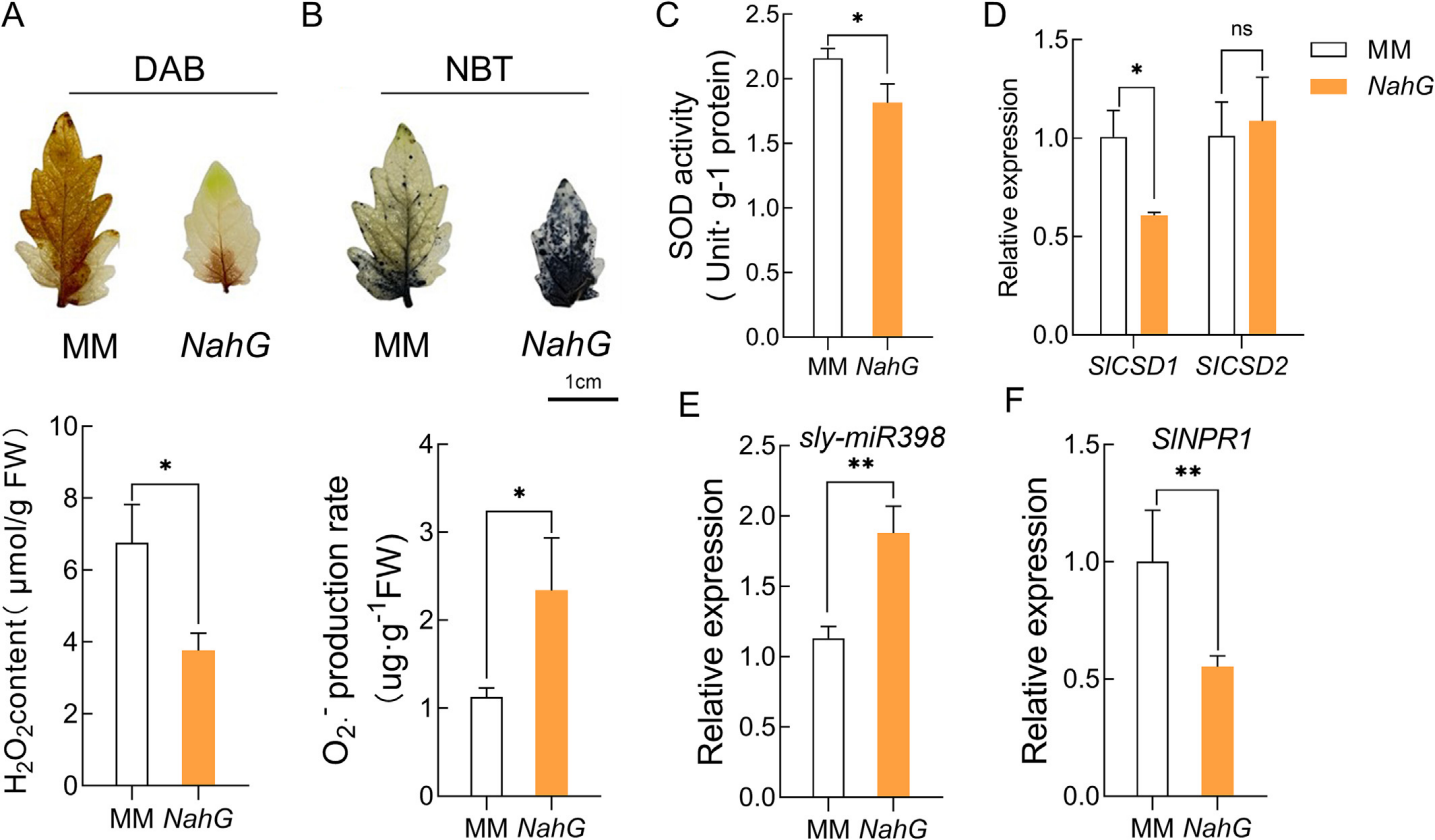
**The accumulation of H_2_O_2_ and expression of *SlCSD1* is inhibited while miR398 is induced in *NahG* plants.** (A-F) H_2_O_2_ visualized using DAB and content (A), O_2_^.-^ visualized using NBT and production rate (B), SOD activity (C), *SlCSD1*, *SlCSD2* expression (D), sly-miR398 expression (E) and *SlNPR1* expression of WT (F) (wild-type, Moneymaker) tomato plants and overexpression of *NahG* transgenic plants were sampled at 28 days under normal condition. Data are presented as the means ± SD (n = 3) The asterisks indicate significant differences based on one-way ANOVA (*P < 0.05, **P < 0.01, ***P < 0.001, ns, no significance).

Our findings revealed a decrease in H_2_O_2_ content and an increase in O_2_^.-^ production rate in *NahG* plants compared to wild-type plants (Fig. 2A, B). Moreover, the activity of SOD and the expression of *SlCSD1* exhibited a decline in *NahG* plants (Fig. 2C, D). Interestingly, the expression of sly-miR398 was found to be up-regulated in *NahG* plants (Fig. 2E). Consistent with our expectations, the expression of *SlNPR1*, encoding the SA receptor NPR1, showed a significant down-regulation in *NahG* plants (Fig. 2F).

### TGA-motif bind directly to the promoter regions of sly-miR398b

**Fig. 3:**
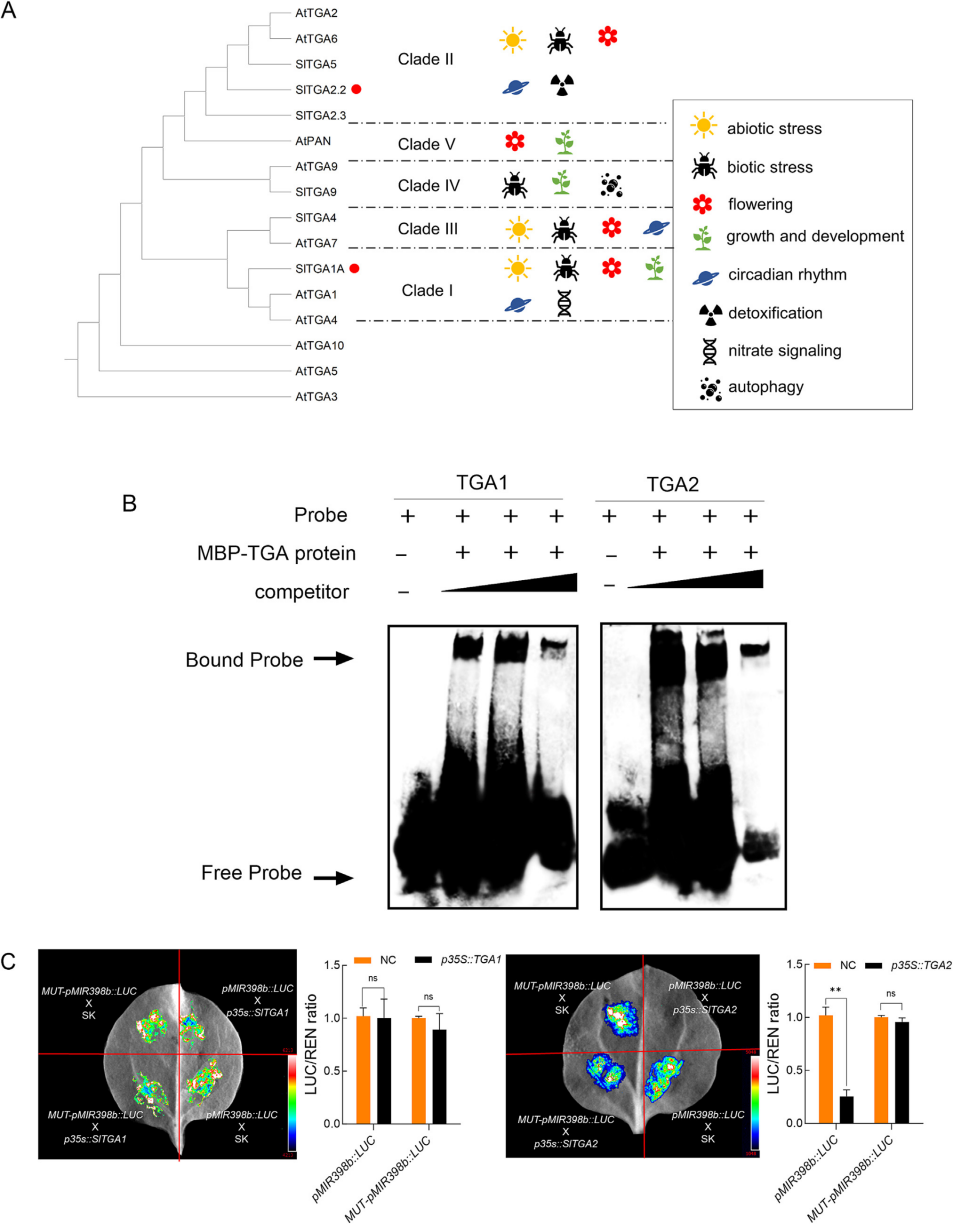
***SlTGA2* inhibited the promoter activity of sly-miR398b.** (A) Phylogenetic tree of the TGA family members in *Arabidopsis thaliana* and *Solanum lycopersicum*, constructed using CLUSTAL X for protein sequence alignment and MEGA X for maximum likelihood analysis. (B) The protein levels of SlTGA1 and SlTGA2 and TGACG-motif-specific binding were detected by Electrophoretic Mobility Shift Assay (EMSA) analysis. Experiments were repeated at least twice with similar results. (C) The constructs containing *pMIR398b::LUC* and *MUT-pMIR398b::LUC* (mutation of 5′-TGACG-3′ to 5′-ACTGC-3′) were separately expressed or co-expressed with *p35S::SlTGA1* or *p35S:: SlTGA2* in *Nicotiana benthamiana* leaves. The ratio of LUC/REN was measured (NC, PG*reen 62-SK* vector). Data are presented as the means ± SD (n = 3) The asterisks indicate significant differences based on one-way ANOVA (*P < 0.05, **P < 0.01, ***P < 0.001, ns, no significance).

To understand the regulation of sly-miR398 by SA, the cis-regulatory elements of the sly-miR398b promoter were analyzed using the online tool PLANTCARE (https://bioinformatics.psb.ugent.be/webtools/plantcare/html/). There were 35 types of cis elements found within a 2.5 kb promoter sequence, with a TGA binding site for SA (5′-TGACG-3′) identified (Supplemental Table S3). Tomato has six TGA genes that are grouped into four classes based on their phylogenetic relationships: Clade I (*SlTGA1*), Clade II (*SlTGA2*, *SlTGA2.3*, and *SlTGA5*), Clade III (*SlTGA4*), and Clade IV (*SlTGA9*) (Fig. 3A).

Branch I and II TGA genes are extensively studied for their roles in defense responses to hormones like salicylic acid (SA) and in abiotic stress and developmental processes. *SlTGA1* and *SlTGA2* were used to detect the interaction between miR398 and SA. Therefore, EMSA experiment was conducted to determine if SlTGA1/2 directly binds to the sly-miR398b promoter. A shifted band was observed when the biotin-labeled DNA fragment containing the core TGA-motif (TGACG) was pre-incubated with mannan-binding protein (Mbp) tagged SlTGA2 protein or SlTGA1 protein, and this binding was outcompeted by the addition of excess unlabeled probes (Fig. 3B). The results suggest that both SlTGA1 and SlTGA2 bind to the miR398b promoter.

To test the hypothesis that SlTGA directly inhibits sly-miR398b transcription, a transient overexpression experiment was conducted. The fluorescence intensity produced by LUC was robustly inhibited by overexpressing *SlTGA2*, but this inhibition was abolished by mutating *SlTGA2* (Fig. 3C). In contrast, overexpressing *p35S::SlTGA1* did not significantly influence miR398b promoter activity. These results suggest that *SlTGA2* may negatively regulate sly-miR398b through binding to TGACG-motif on the upstream promoter.

### Sly-miR398b affected the SA accumulation

**Fig. 4:**
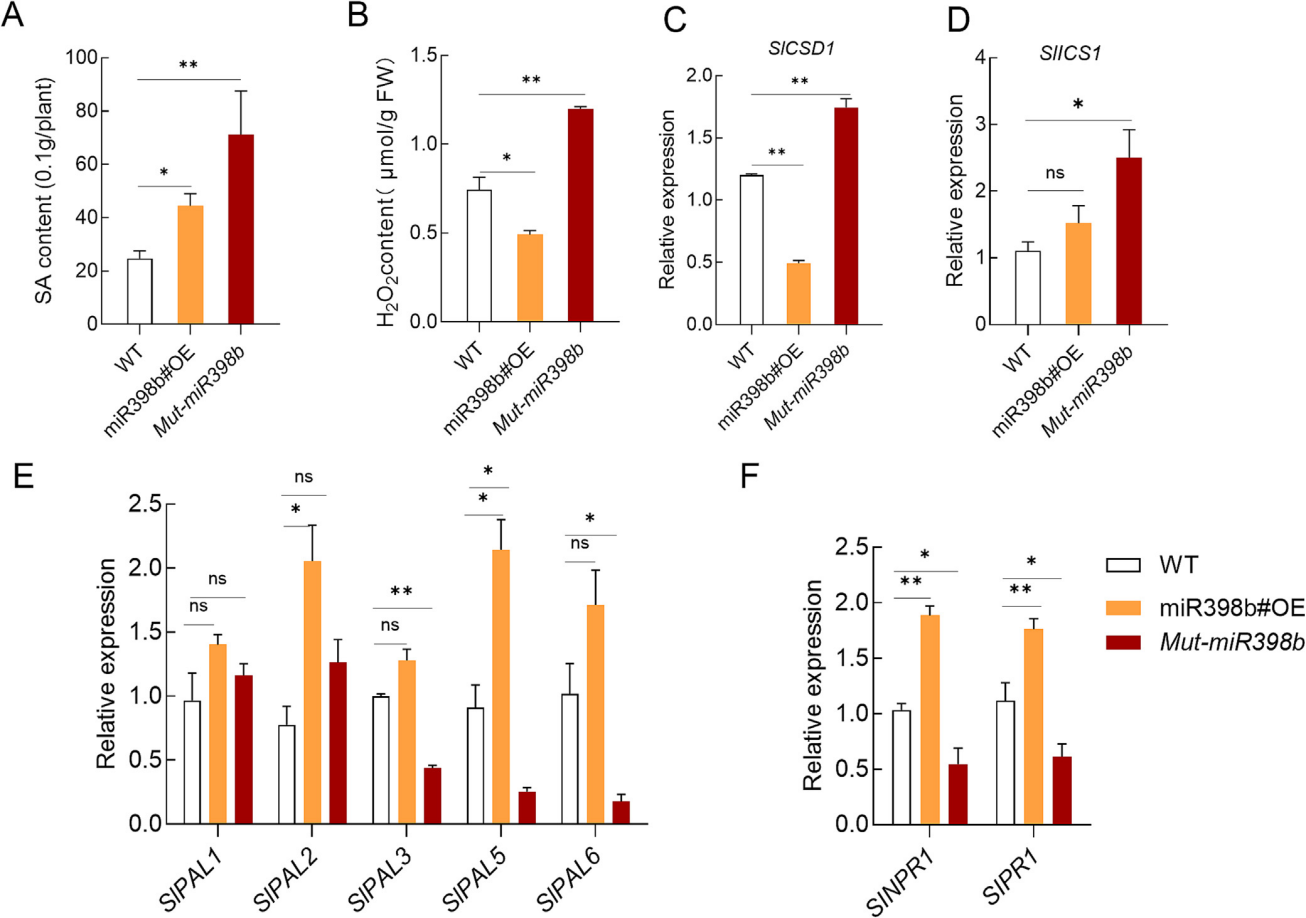
**Sly-miR398b positively affected the synthesis and signaling of SA.** (A-F) SA content (A), H_2_O_2_ content (B), *SlCSD1* expression (C), *SlICS1* expression (D), *SlPAL1, 2, 3, 5*,6 expression (E) and *SlNPR1, SlPR1* expression (F) was measured in the leaves of WT, miR398b#OE and *Mut-miR398b* plants, which grown under normal condition for 28 days. Data are presented as the mean ± SD (n = 3). The asterisks indicate significant differences based on one-way ANOVA (*P < 0.05, **P < 0.01, ***P < 0.001, ns, no significance).

To explore the role of miR398 in SA synthesis and signalling, transgenic tomato plants that overexpressed sly-miR398b and plants with a loss of function mutation in *Mut-miR398b* were generated. It was found that both the overexpression of sly-miR398b and the loss of function mutation increased the levels of SA in the plants, compared to the wild-type plants. The increase in SA levels was more pronounced in *Mut-miR398b*, where the levels were 4-fold higher than in the wild-type plants. On the other hand, the overexpression of sly-miR398b resulted in a 1-fold increase in SA levels (Fig. 4A).

It was also found that the overexpression of sly-miR398b reduced the levels of H_2_O_2_ compared to the wild-type plants. However, the loss of function mutation in sly-miR398b increased the levels of H_2_O_2_ and the expressions of *SlCSD1* dramatically (Fig. 4B, C). Our results also revealed changes in the expression of key genes involved in SA synthesis, such as *PHENYLALANINE AMMONIA 2/3/5/6 (SlPAL2*/*3*/*5*/*6)* which were up-regulated in the sly-miR398b#OE plants (Fig. 4E). On the other hand, in *Mut-miR398b* plants the expression of *ISOCHORISMATE SYNTHASE 1(SlICS1)* and *SlPAL2* were induced, and *SlPAL3*, *SlPAL5* and *SlPAL6* were inhibited (Fig. 4D, E).

Additionally, the expression of *SlNPR1* (a key receptor in SA signaling), was up-regulated in the sly-miR398b#OE plants, while it was down-regulated in *miR398b* plants (Fig. 4F). Meanwhile, the SA response gene *SlPR1* also showed a similar trend with *SlNPR1* (Fig. 4F)*.*

### The induce of H_2_O_2_ by SA is partially dependent on *SlCSD1*

**Fig. 5:**
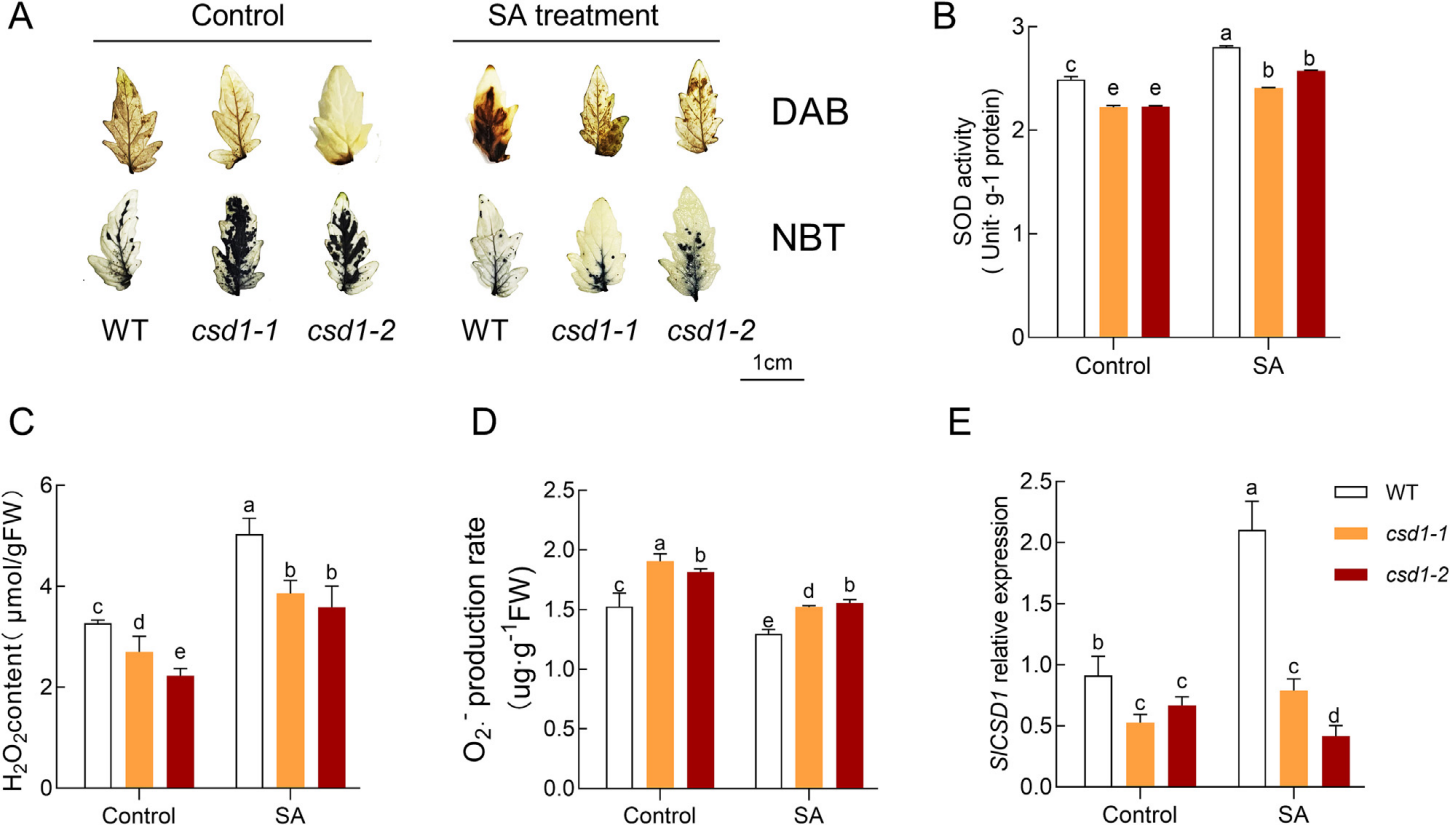
**H_2_O_2_ signaling induced by SA is dependent on *SlCSD1*.** A-E, H_2_O_2_ and O_2_^.-^ visualized using DAB and NBT (A), SOD activity (B), H_2_O_2_ content (C), O_2_^.-^ production rate (D) and *SICSD1* expression (E) treated with SA (0.01 mM) of WT (wild-type, Ailsa Craig) and *SICSD1*-silenced transgenic tomato plants for 2 day. Data are presented as the mean ± SD (n = 3). The statistical analysis of was based on one-way ANOVA. Different letters above the bars indicate significant differences (*P < 0.05*).

To determine the dependence of SA-mediated ROS metabolism on *SlCSD1*, two loss-of-function mutants of *SlCSD1* (cs*d1-1*, *csd1-2*) were generated and exposed them to SA treatment. Under control conditions, the content of H_2_O_2_ was reduced, and the production rate of O_2_^.-^ was increased in both *csd1* lines compared to wild-type plants (Fig. 5A). Similar to the results in Fig. 1, exposure to 0.01 mM SA promoted the accumulation of H_2_O_2_. However, the increase in H_2_O_2_ content and decrease in O_2_^.-^ production rate caused by exogenous SA was partially abolished by the *SlCSD1* mutation (Fig. 5C, D). Additionally, the activity of SOD and the relative expression of *SlCSD1* were reduced in both *csd1* lines compared to WT (Fig. 5B, E).

**Fig. 6:**
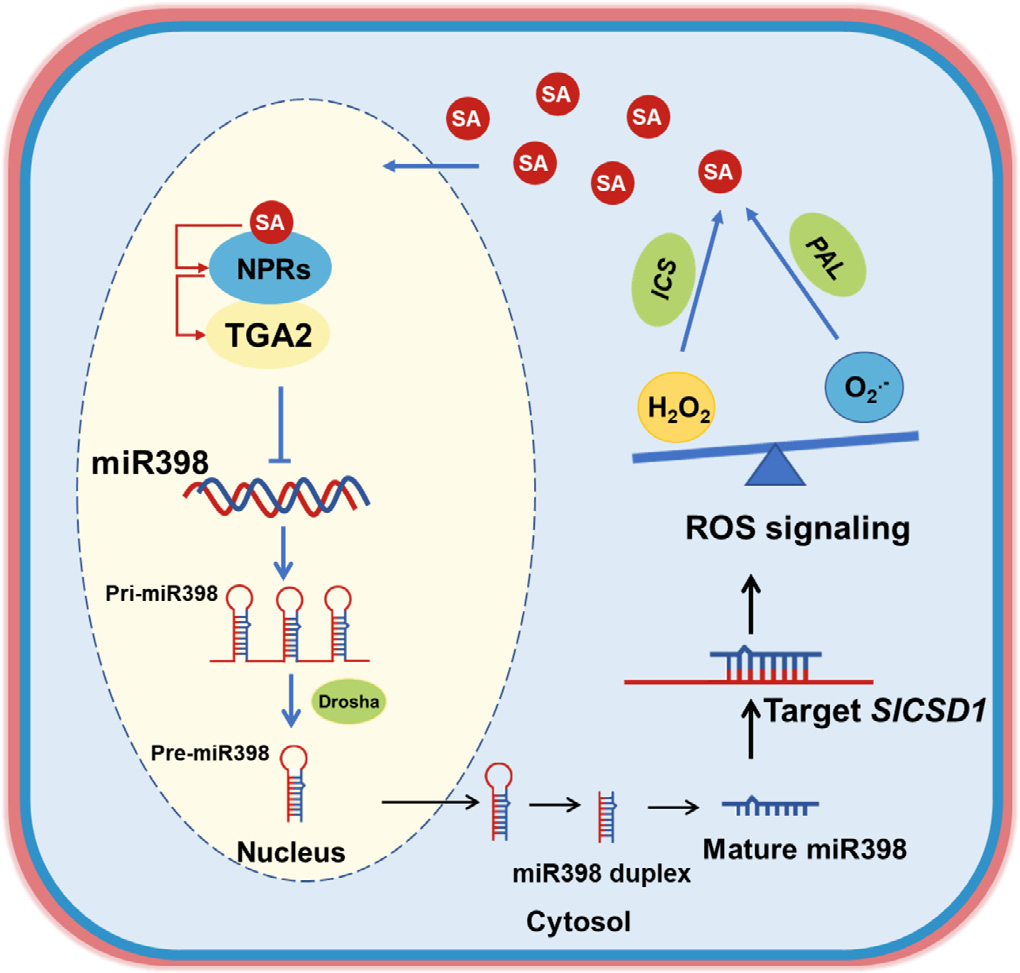
**Proposed model for the role of miR398-SlCSD1 module in SA-H_2_O_2_ amplifying feedback loop.** When SA levels are elevated, *SlTGA2* transcription factor together with NPRs represses the transcriptional activity of sly-miR398b, thereby releasing the inhibition of its target *SlCSD1*. The up-regulation of *SlCSD1* expression led to enhanced SOD activity and increased the content of H_2_O_2_, which induced SA synthesis via the isochorismate synthase (ICS) pathway. Additionally, superoxide promoted SA synthesis via the phenylalanine ammonia-lyase (PAL) pathway.

## Discussion

Both SA and ROS are key players involved in plant immunity responses and they are supposed to form a feed-forward loop that regulates SA signalling. However, the precise mechanism by which SA regulates ROS signalling is poorly understood. In this study, the miR398-SlCSD1 module was identified as a potential mediator between SA and ROS.

### SA induces H_2_O_2_ signalling via the miR398-SlCSD1 module mediated by TGA2

Our results demonstrate that SA induces H_2_O_2_ accumulation in a dose- and time-dependent manner. Specifically, low concentrations of SA (0.01 mM and 0.1 mM) and short-term treatments (6 h to 2d) promoted H_2_O_2_ accumulation, while higher concentrations of SA (1 mM) and prolonged exposure (7 d) led to reduced H_2_O_2_ levels (Fig. 1A, 1B, and Supplemental Fig. S2A, C). In addition, excessive doses of SA can induce cell death and lethal ROS accumulation within a few hours, resulting in inhibited plant growth and development. It is noteworthy that SA has been reported to enhance H_2_O_2_ scavenging under various stressful conditions in the long round (from 1 week to 2 weeks), including salinity [20], UV-B radiation [44], ozone [45], and high temperature [46]. The initial increase in H_2_O_2_ induced by SA (6 h to 2d) may function as a signalling molecule, triggering antioxidant responses and contributing to ROS scavenging under both biotic and abiotic stresses.

However, at higher SA concentrations (1 mM, Fig. 1D), SOD activity declined, likely due to oxidative stress caused by excessive ROS accumulation (O_2_^.-^ Fig. 1C). In addition to *SlCSD1*, the expression of *Mn-SOD* and *Fe-SOD1* was also downregulated at 1 mM SA (Supplemental Fig. S2D), further contributing to the reduced SOD activity.

Concurrently, it was observed that the expression of sly-miR398b and *SlCSD1* responded to changes in SA in opposite directions, with sly-miR398b being down-regulated and *SlCSD1* being up-regulated (Fig. 1E, 1H). Additionally, the reduction of SA in *NahG* plants decreased the activity of SOD, down-regulated the expression of *SlCSD1*, and up-regulated the expression of sly-miR398b (Fig. 2C–E), suggesting that *SlCSD1* is transcriptionally inhibited by sly-miR398b. However, the expression of *SlCSD2* was not affected by reduced SA in *NahG* plants, as it is primarily translationally repressed by miR398. Besides, in *NahG* plants, SA is converted into catechol, which scavenges ROS (both O_2_^.-^and H_2_O_2_) directly. However, the changes of H_2_O_2_ and O_2_^.-^ exhibited opposite opposition in *NahG* plants compared with WT plants (Fig. 2A, B). Thus the induce of O_2_^.-^ and reduction of H_2_O_2_ in *NahG* plants was due to the decrease of endogenous SA but not the increase of catechol contents.

Furthermore, the mutation of sly-miR398b and *SlCSD1* resulted in opposite responses to SA. SA-treated *miR398b* plants exhibited relative higher H_2_O_2_ content compared to the wild-type under the same SA concentration (Supplemental Fig. S3A, B). Conversely, the increase of H_2_O_2_ by SA was partially diminished in both *csd1* lines, accompanied by decreased activity of SOD (Fig. 5), indicating that H_2_O_2_ accumulation by SA is dependent on *SlCSD1*.

Importantly, SlTGA2 was identified as a mediator of the regulation of the miR398b-SlCSD1 module by SA. TGA transcription factors, interacting with NPR cofactors, are essential for the SA mediated defense responses [47]. In the present study, we identified six TGA genes in the tomato genome, and discovered a specific TGA binding site (TGACG-motif) upstream of the sly-miR398b promoter. SlTGA2, induced by SA (Fig. S2E), directly bound to the sly-miR398b promoter and inhibited its activity (Fig. 3B, Fig. 3C). Notably, recent studies have demonstrated that clade II TGAs directly activate the expression of *RBOHD* and *RBOHF*, contributing to SA-induced ROS production [48]. Additionally, the interaction between two type II TGAs, *TGA2.1* and *TGA2.3*, activates the expression of *StPRX07*, a peroxidase member (StPRX), affecting apoplastic ROS production in potato [49]. These observations underline TGA2′s central role in coordinating ROS dynamics. It facilitates O_2_^.-^ production via activation of RBOHD and RBOHF, promotes its conversion to H_2_O_2_ by repressing miR398, and enables scavenging of excess H_2_O_2_ through peroxidase activation.

In addition to SOD, the reduced activity of catalase (CAT) and ascorbate peroxidase (APX) also contributes to SA-induced H_2_O_2_ accumulation [9,21] Analysis of the *SlCAT1* (*Solyc12g094620.1*) and *SlAPX6* (*Solyc11g018550.4.1*) promoters revealed the presence of TGA binding sites, including one in the *SlCAT1* promoter (Supplemental Table S4 and Table S5). Further studies are needed to investigate the interaction between *SlTGA2* and *SlCAT1* to clarify how SA regulates *SlCAT1* expression.

In general, SA interacts with TGAs transcription factors through NPRs receptors, which in turn directly induces the response of downstream defense genes [23,50]. Our results reveal that SA directly represses miR398 through the transcription factor TGA2 to induce the expression of downstream defense genes such as *SlPR1*, *SlCSD* and *SlPOD*, providing new insights into the signalling pathway transduction of SA.

### Fluctuations in miR398 level induce SA synthesis

SA is synthesized in plants through two main pathways, the isochorismate synthase (ICS) pathway and the phenylalanine ammonia-lyase (PAL) pathway [51]. In the present study, it was observed that both overexpression and knockdown of sly-miR398b led to increased SA contents via the PAL and ICS pathways, respectively (Fig. 4A–D), highlighting the important role of miR398b in regulating SA biosynthesis. Notably, in sly-miR398b overexpression plants, the upregulation of *SlNPR1* expression was observed compared to WT plants (Fig. 4C). This observation is consistent with previous studies that have reported an upregulation of *NPR1* in response to elevated levels of SA [52,53].

Interestingly, knockdown of sly-miR398b resulted in a more substantial increase in SA content compared to its overexpression (Fig. 4A), coinciding with the activation of the ICS pathway. The ICS pathway, primarily occurring in the chloroplast, contributes up to 95 % of total SA biosynthesis in plants [54]. The elevated expression of *SlICS1* in *Mut-miR398b* plants was accompanied by increased H_2_O_2_ levels (Fig. 4B), suggesting a potential link between H_2_O_2_ signalling and SA synthesis. Recent studies have shown that H_2_O_2_ promotes *ICS1* expression by sulfenylating the transcription factor CCA1 HIKING EXPEDITION (CHE), enhancing its binding to the *ICS1* promoter [55]. This mechanism could underlie the observed induction of *SlICS1* in *Mut-miR398b* plants. Additionally, the downregulation of *SlNPR1* in *Mut-miR398b* plants (Fig. 4F) may result from a negative feedback loop. Elevated SA levels are known to facilitate NPR1 degradation via the 26S proteasome pathway through interactions with NPR3 [55]. This feedback regulation could explain the reduced *SlNPR1* expression despite increased SA accumulation in *Mut-miR398b* plants.

The miR398-SlCSD1 module also plays a pivotal role in SA-mediated salt tolerance. Under salt stress combined with SA treatment, sly-miR398b overexpression plants exhibited greater dry mass accumulation (Supplemental Fig. S5A and S5B) and reduced oxidative damage (Supplemental Fig. S5C) compared to wild-type plants. Moreover, the enhanced expression of *SlNPR1* in sly-miR398b overexpression plants (Supplemental Fig. S5G) further supports the positive regulatory role of miR398 in SA signalling. These findings highlight the significance of the miR398-SlCSD1 module in SA-mediated stress responses and provide novel insights into its molecular mechanisms.

While our findings strongly support the role of the miR398–SlCSD1 module in SA–H_2_O_2_ signalling under exogenous SA application, its functional involvement in modulating endogenous SA biosynthesis or signalling remains to be fully elucidated. Future work under biotic stress conditions, such as pathogen challenge, will be essential to determine whether this module can regulate endogenous SA dynamics in a natural physiological context.

## Conclusion

Based on the current data, we propose a working model for the role of the miR398-SlCSD1 module in SA-H_2_O_2_ amplifying feedback loop (Fig. 6). When SA levels are elevated, the TGA2 transcription factor, in conjunction with NPR1, may suppress sly-miR398b transcription, thereby releasing the repression on its targets, *SlCSD1*. The increased expression of *SlCSD1* results in the augmented activity of SOD, leading to the production of H_2_O_2_, which in turn induces the synthesis of SA via the ICS pathway. Additionally, overexpression of sly-miR398b was associated with elevated superoxide levels and increased SA content, potentially through activation of the PAL pathway. While these findings suggest a regulatory role for miR398 in modulating SA–ROS signalling, further work, particularly under endogenous SA-inducing conditions such as pathogen challenge, is needed to validate this model. Nonetheless, our results provide a useful framework for future efforts to enhance crop stress tolerance through the fine-tuning of microRNA-mediated signalling networks.

## Accession numbers

The mature sequence of MIR398 in this study was miR398b: UUGUGUUCUCAGGUCACCCCU, miR398a: UAUGUUCUCAGGUCGCCCCUG, miR398c: UGUGUUCUCAGGUUACCCCUG. Sequence data used in this article can be found in the *Solanum lycopersicum* Information Resource (https://solgenomics.net/) under the following accession numbers: *SlCSD1* (Solyc01g067740.3.1), *SlCSD2* (Solyc11g066390.2.1), *SlTGA1* (Solyc10g080770.3.1), *SlTGA2* (Solyc04g011670.3.1), *SlNPR1* (Solyc10g080770.1.1), *SlPR1* (Solyc01g106620.2.1), *SlICS1* (Solyc06g071030.3.1), *SlPAL1* (Solyc09g007900.4.1), *SlPAL2* (Solyc05g056170.3.1), *SlPAL3* (Solyc09g007920.4.1), *SlPAL5* (Solyc09g007910.4.1), *SlPAL6* (Solyc10g086180.2.1), *SlCAT1* (Solyc12g094620.1) and *SlAPX6* (Solyc11g018550.4.1).

## Declaration of competing interest

The authors declare that they have no known competing financial interests or personal relationships that could have appeared to influence the work reported in this paper.
