# Supplementary material for: miR398-SlCSD1 module participates in the SA-H2O2 amplifying feedback loop in Solanum lycopersicum
Source: J Adv Res. 2025 Apr 22;80:19–30. doi: 10.1016/j.jare.2025.04.035 (PMC12869259; doi:10.1016/j.jare.2025.04.035)
Supplement: Supplementary Data 1 [file mmc1.docx]

**Supplemental information**

**miR398-SlCSD1 module participates in the SA-H_2_O_2_ amplifying feedback loop**

**in *Solanum lycopersicum***

Xiujuan Wang, Xinshan Zhang, Yuanyuan Liu, Lei Ru, Guochao Yan, Yunmin Xu, Youjian Yu, Zhujun Zhu*, Yong He*


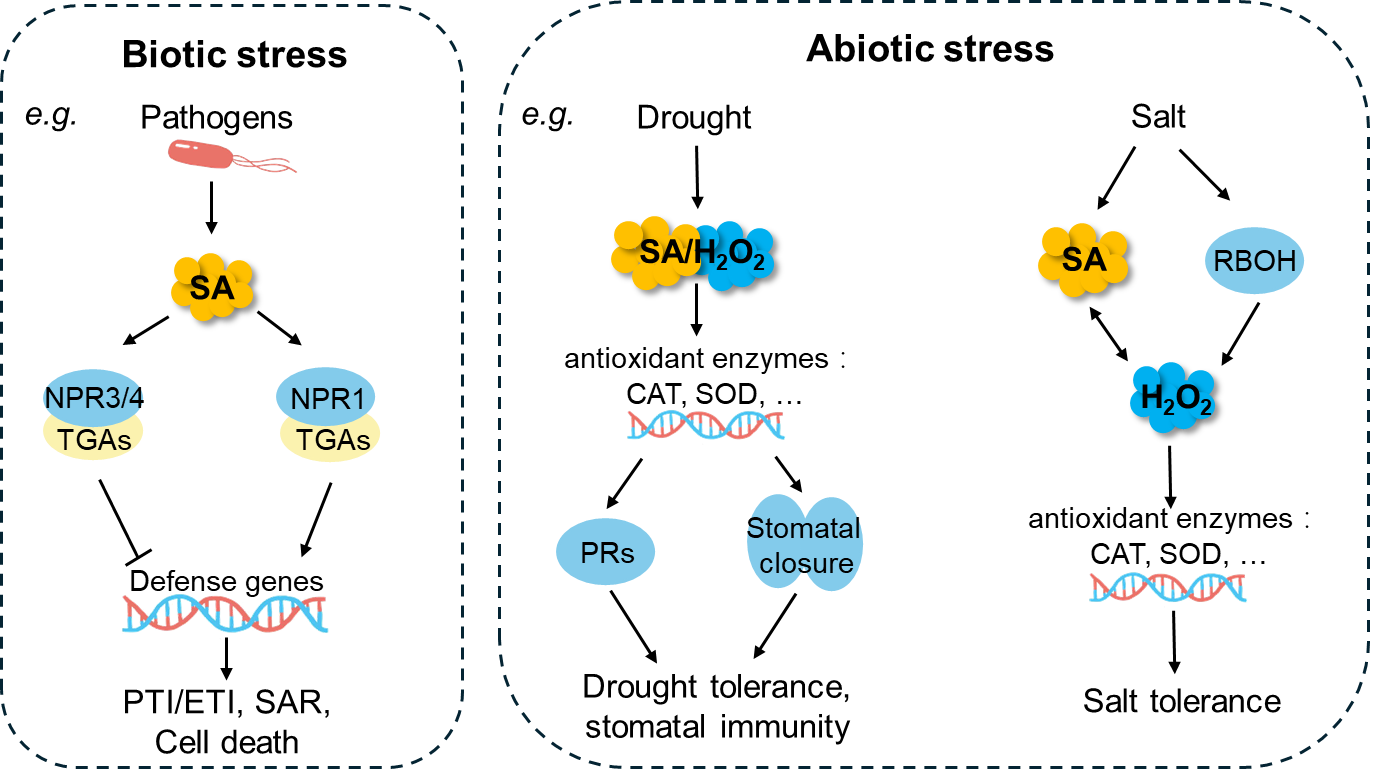


**Figure S1. The regulatory mechanism of Salicylic Acid (SA) under Biotic and Abiotic Stresses [1-5].** SA: Salicylic Acid, H_2_O_2_: hydrogen peroxide, NPR1: Non-expressor of Pathogenesis-Related genes 1, NPR3/4: Non-expressor of Pathogenesis-Related genes 3/4, TGAs: TGACG Motif Binding Factor (or TGA Transcription Factors), PTI: Pattern-Triggered Immunity, ETI: Effector-Triggered Immunity, SAR: Systemic Acquired Resistance, CAT: Catalase, SOD: Superoxide Dismutase, RBOH: Respiratory Burst Oxidase Homolog, PR: Pathogenesis-Related (genes or proteins).

**Figure S2. The effect of SA on H_2_O_2_ and SOD levels under different time and concentration conditions.**

Four-week-old plants of 'Micro-Tom' were treated under normal condition and SA (0.01 mM, 0.1 mM, 0.5 mM and 1 mM).

(A, B) The content of H_2_O_2_ and SOD activity in leaves were determined after 6h, 1, 2, 3, and 7 days of 0.01 mM SA treatment.

(C) The content of H_2_O_2_ in leaves were determined after 2 days of treatment.

(D) The expression of *Mn-SOD*, *Fe-SOD1* and *Fe-SOD2* in leaves were determined after 2 days of 1 mM SA treatment.

(E) The expression of *SlTGA1 and SlTGA2* in leaves were determined after 2 days of 0.01mM SA treatment.

**Figure S3. sly-miR398b positively affected the content of H_2_O_2_, the expression of *SlGPOD*, *SlSADR1, SlPAD4*，*SlEDS1* and *SlCSD1.***

(A-C) H_2_O_2_ visualized with DAB (A), H_2_O_2_ content (B), *SlGPOD* expression (C) were treated under normal condition and SA (0.01 mM) of WT (‘Micro-Tom’), miR398b#OE and *Mut-miR398b* plants

(D) Real-time quantification of *SlSADR1, SlPAD4*，*SlEDS1* and *SlCSD1* expression in four weeks old plant leaves of WT, miR398b#OE and *Mut-miR398b* plants, with *SIACTIN* serving as an internal reference.


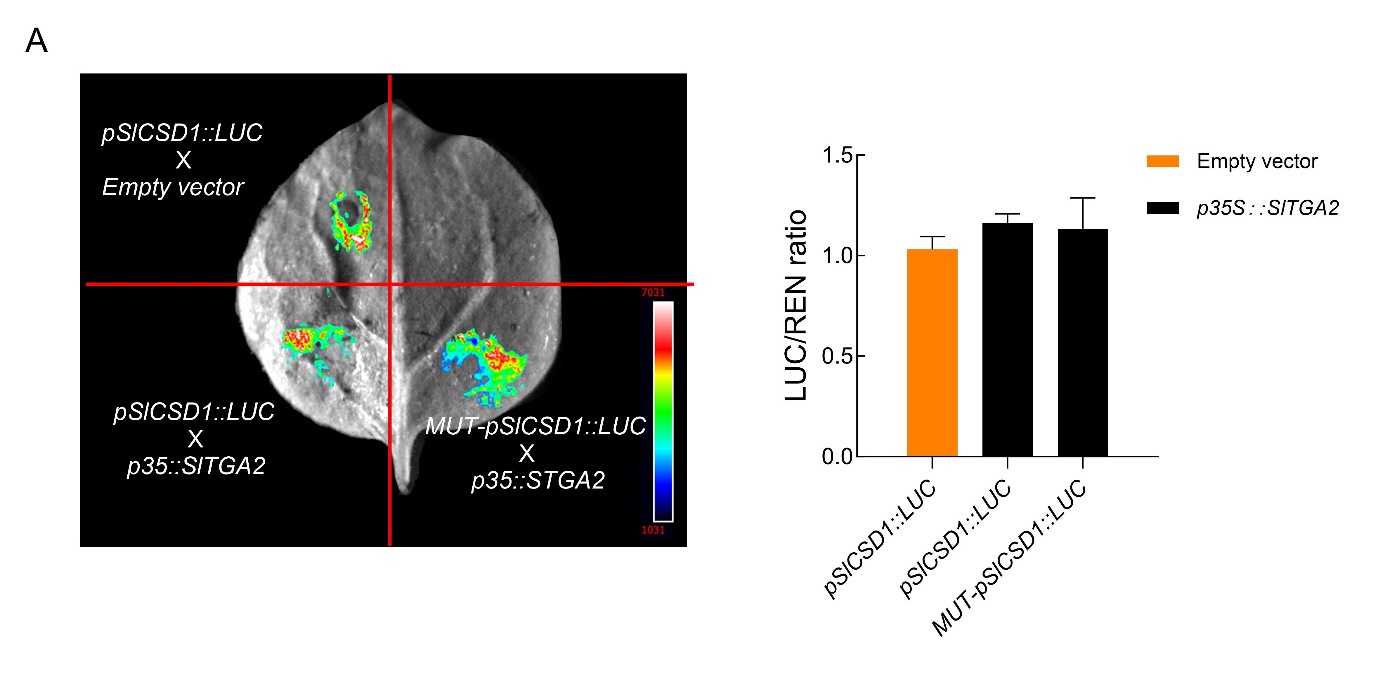


**Figure S4. *SICSD1* does not interact with *SlTGA2***

The constructs containing *pSlCSD1::LUC* or *MUT- pSlCSD1::LUC* were separately expressed or co-expressed with *p35S::SlTGA2* (A) in *Nicotiana benthamiana* leaves using Agrobacterium-mediated infiltration at the indicated OD6_00_ value. CCD images revealing the fluorescence intensities of *pSlCSD1::LUC* or *MUT- pSlCSD1::LUC* were taken with an Tanon Fully Automated Chemiluminescence/Fluorescence Image Analysis System. The ratio of LUC/REN was measured. Data are presented as the means ± SD (n = 3) The asterisks indicate significant differences based on one-way ANOVA (**P < 0.05, **P < 0.01, ***P < 0.001*, ns, no significance).

**Figure S5. The response of the miR398-SlCSD1 module under salt stress.**

Tomato seedlings (25 d after germination) of WT and miR398b#OE were subjected to four different treatments (normal conditions, 0.01 mM SA, 100 mM NaCl, and 0.01 mM SA+100 mM NaCl) for 7 days with four replicates for each treatment.

(A) Typical plant phenotype of WT and miR398b#OE, (B) above-ground dry mass of plants, (B) above-ground fresh mass of plants (D) MDA content, (E) miR398 expression, (F) SOD activity, (G) *SlCSD1* expression, (H) *SlNPR1* expression. The data are the mean values ± SE of four individual replicates and different letters indicate significant differences (p < 0.05).

**Table S1.** Primer used for cloning, EMSA and qRT-qPCR assay

| *Gene* | Use | Direction | Sequence (5‘ to 3’) |
| --- | --- | --- | --- |
| *Pri-miR398b* | Cloning | Forward | TGACCACATTGTGATGCTCTG |
|  |  | Reverse | CCCTTTTAGTGCATCGAACTG |
| *Sly-miR398b* | CRISPR/Cas9 | SgRNA | AATCCCCAGAGGAGTGTGCC |
| *csd1* | CRISPR/Cas9 | SgRNA | GCCGTCCTTAACAGCAGTGA |
| *SICSD1* | Cloning | Forward | GAACTCATCATTATCAGCAC |
|  |  | Reverse | ACCACAAGCAATCCTTCC |
| *SICSD2* | Cloning | Forward | CACTCAATCTTCACCACAAC |
|  |  | Reverse | CAATCGTCCACCAGCATT |
| *pMIR398b* | Cloning | Forward | CGAGTGATAACATGGCAC |
|  |  | Reverse | ATATAACCTATAGTTTAATATTCC |
| *TGA1* | Cloning | Forward | CAAGTTGGGATGGTTGCACA |
|  |  | Reverse | GGCAAAACTCGTCGAATGGT |
| *TGA2* | Cloning | Forward | ACTGAGGTTGTGGATTGTTGA |
|  |  | Reverse | CCGGCCAGTGTATCGAAGTA |
| *miR398* | RT-qPCR | Stem loop primer | GTCGTATCCAGTGCAGGGTCCGAGGTATTCGCACTGGATACGACAGGGGT |
|  |  | Forward | GCGCGTTGTGTTCTCAGGTC |
|  |  | Reverse | AGTGCAGGGTCCGAGGTATT |
| *Pri-miR398a* | RT-qPCR | Forward | TGTGGATGTGTTCAATAGGGTTG |
|  |  | Reverse | ATAGTCTACAGGGGCGACCT |
| *Pri-miR398b* | RT-qPCR | Forward | CTCATTTGTGTTGATAGAATGACC |
|  |  | Reverse | GGGGATTCACCATTGCTCAT |
| *Pri-miR398c* | RT-qPCR | Forward | GGTGGAGGTATTCAACAGGG |
|  |  | Reverse | GAAGCAAATCGCCACCAAGT |
| *SlCSD1* | RT-qPCR | Forward | GGTGTTAGTGGCACCATCCT |
|  |  | Reverse | AGCACCATGCTCCTTACCAG |
| *SlCSD2* | RT-qPCR | Forward | ACATTGTTGCTGGTCCTAATGAGA |
|  |  | Reverse | CCCAATAACGCCTCTTCCCA |
| *SlNPR1* | RT-qPCR | Forward | GCAACACGAAGCTGGAACTC |
|  |  | Reverse | TGAACCATGAAGGCCACAGC |
| *SIPR1* | RT-qPCR | Forward | CGTCTTGGTTGTGCTAGGGT |
|  |  | Reverse | TGGACGTTGTCCTCTCCAGT |
| *SlICS1* | RT-qPCR | Forward | ACTTGAGGCACCTCTTTTGAC |
|  |  | Reverse | AGGTTTTTGATGCTCTGTGCT |
| *SlPAL1* | RT-qPCR | Forward | TCATCCAGCAAGATTCAGCGA |
|  |  | Reverse | AGTTGGAGCTGCAAGGATCA |
| *SlPAL2* | RT-qPCR | Forward | TGGTACAGCTGTTGGCTCTG |
|  |  | Reverse | CCGTAAATTCGGGCTTCCCT |
| *SlPAL3* | RT-qPCR | Forward | GGACGAATTAAAGGCCGTGT |
|  |  | Reverse | TAGGAATTGCAGGGTTGCCA |
| *SlPAL5* | RT-qPCR | Forward | GCTACAAGTGGTGGAAAGGGA |
|  |  | Reverse | AGGACTTGTCTCAGCTTCTGC |
| *SlPAL6* | RT-qPCR | Forward | TGCTAATGTGCTCGCGGTAT |
|  |  | Reverse | TCAGTGAACTCGGGCTTTCC |
| *Mn-SOD* | RT-qPCR | Forward | TGGAGAACCCAAAAGTTGCAG |
|  |  | Reverse | TCGACATACGCCCTGTGATG |
| *Fe-SOD1* | RT-qPCR | Forward | AAAGGGTTCTCTTGGCTGGG |
|  |  | Reverse | GGGTCCTGATTTGCAGTGGT |
| *Fe-SOD2* | RT-qPCR | Forward | AGATGAAGCCTAACGGAGGA |
|  |  | Reverse | GTTTGTATGCAAGCCAGGAC |
| *SlU6* | RT-qPCR | Forward | CATCCGATAAAATTGGAACGA |
|  |  | Reverse | TTTGTGCGTGTCATCCTTGCG |
| *SlACTIN* | RT-qPCR | Forward | TTCAAAGGGCGAGTACGACG |
|  |  | Reverse | ACTTGCCTAACAGCAGACCC |
|  |  |  |  |

**Table S2.** Prediction of cis-acting elements of the 1.5k bp *SICSD1* promoter

| **Regulatory components** | **Sequence** | **Function** |
| --- | --- | --- |
| ABRE | CACGTG, ACGTG | Cis-acting element involved in the abscisic acid responsiveness |
| ACE | CTAACGTATT | Cis-acting element involved in light responsive |
| AE-box | AGAAACTT | Part of module for light response |
| ARE | AAACCA | Cis-acting regulatory element essential for the anaerobic induction |
| AT1-motif | AATTATTTTTTATT | Part of a light responsive module |
| Box 4 | ATTAAT | Part of a conserved DNA module involved in light responsiveness |
| CAAT-box | CAAAT, CAAT | Common cis-acting element in promoter and enhance regions |
| ERE | ATTTCATA | Ethylene-responsive element |
| G-box | CACGAC, TACGTG, CACGTG | Cis-acting regulatory element involved in light responsiveness |
| GATA-motif | GATAGGA | Part of a light responsive element |
| MYC | CATTTG |  |
| MYb | CAACTG, TAACTG |  |
| MYc | TCTCTTA |  |
| TATA | TATAAAAT |  |
| TATA-box | TATAA, TAAAGATT, TACAAA, | Core promter element around -30 of transcription start |
| TCA | TCATCTTCAT |  |
| TCCC-motif | TCTCCCT | Part of a light responsive element |
| W box | TTGACC | Fungal elicitor responsive element |
| Chs-CMA1a | TTACTTAA | Part of a light responsive element |

**Table S3.** Prediction results of cis-acting elements of sly-miR398b promoter

| **Regulatory components** | **Sequence** | **Function** |
| --- | --- | --- |
| ABRE | CACGTG, ACGTG | Cis-acting element involved in the abscisic acid responsiveness |
| ACE | CTAACGTATT | Cis-acting element involved in light responsive |
| AE-box | AGAAACTT | Part of module for light response |
| ARE | AAACCA | Cis-acting regulatory element essential for the anaerobic induction |
| AT1-motif | AATTATTTTTTATT | Part of a light responsive module |
| Box 4 | ATTAAT | Part of a conserved DNA module involved in light responsiveness |
| CAAT-box | CAAAT, CAAT | Common cis-acting element in promoter and enhance regions |
| CGTCA-motif | CGTCA | Cis-acting regulatory element involved in the MeJA-responsiveness |
| DRE core | GCCGAC |  |
| ERE | ATTTCATA | Ethylene-responsive element |
| G-BOX | CACGTG | Cis-acting regulatory element involved in light responsiveness |
| G-box | CACGAC, TACGTG, CACGTG | Cis-acting regulatory element involved in light responsiveness |
| GATA-motif | GATAGGA | Part of a light responsive element |
| GT1-motif | GGTTAA | Light responsive |
| MBS | CAACTG | MYB biding site involved in drought inducibility |
| MYC | CATTTG |  |
| MYb | CAACTG, TAACTG |  |
| MYc | TCTCTTA |  |
| O2-site | GATGACATGG | Cis-acting regulatory element involved in zein metabolism regulatory |
| TATA | TATAAAAT |  |
| TATA-box | TATAA, TAAAGATT, TACAAA, taTATAAAtc | Core promter element around -30 of transcription start |
| TCA | TCATCTTCAT |  |
| TCCC-motif | TCTCCCT | Part of a light responsive element |
| TGACG-motif | TGACG | Cis-acting regulatory element involved in the MeJA-responsivenss |
| W box | TTGACC | Fungal elicitor responsive element |
| Chs-CMA1a | TTACTTAA | Part of a light responsive element |

**Table S4.** Prediction results of cis-acting elements of *SlCAT1* promoter

| **Regulatory components** | **Sequence** | **Function** |
| --- | --- | --- |
| AE-box | AGAAACTT | Part of module for light response |
| ARE | AAACCA | Cis-acting regulatory element essential for the anaerobic induction |
| Box 4 | ATTAAT | Part of a conserved DNA module involved in light responsiveness |
| CAAT-box | CAAAT, CAAT | Common cis-acting element in promoter and enhance regions |
| CGTCA-motif | CGTCA | Cis-acting regulatory element involved in the MeJA-responsiveness |
| ERE | ATTTCATA | Ethylene-responsive element |
| GA-motif | ATAGATAA | Cis-acting regulatory element involved in light responsiveness |
| GATA-motif | GATAGGA | Part of a light responsive element |
| GT1-motif | GGTTAA | Light responsive |
| MYC | CATTTG, TCTCTTA |  |
| MYb | CAACTG, TAACTG |  |
| TATA | TATAAAAT |  |
| TATA-box | TATAA, TAAAGATT, TACAAA, taTATAAAtc | Core promter element around -30 of transcription start |
| P-box | CCTTTTG | gibberellin-responsive element |
| TCA-element | TCATCTTCAT | cis-acting element involved in salicylic acid responsiveness |
| TCCC-motif | TCTCCCT | Part of a light responsive element |
| TGACG-motif | TGACG | Cis-acting regulatory element involved in the MeJA-responsivenss |
| Chs-CMA1a | TTACTTAA | Part of a light responsive element |

**Table S5.** Prediction results of cis acting elements of *SlAPX6* promoter

| **Regulatory components** | **Sequence** | **Function** |
| --- | --- | --- |
| ABRE | CACGTG, ACGTG | Cis-acting element involved in the abscisic acid responsiveness |
| ARE | AAACCA | Cis-acting regulatory element essential for the anaerobic induction |
| Box 4 | ATTAAT | Part of a conserved DNA module involved in light responsiveness |
| ERE | ATTTCATA | Ethylene-responsive element |
| G-BOX | CACGTG | Cis-acting regulatory element involved in light responsiveness |
| HD-zip 1 | CAAT(A/T) ATTG | element involved in differentiation of the palisade mesophyll cells |
| GT1-motif | GGTTAA | Light responsive |
| MBS | CAACTG | MYB biding site involved in drought inducibility |
| MYC | CATTTG |  |
| MYb | CAACTG, TAACTG |  |
| TATA | TATAAAAT |  |
| TATA-box | TATAA, TAAAGATT, TACAAA, taTATAAAtc | Core promter element around -30 of transcription start |
| TATC-box | TATCCCA | cis-acting element involved in gibberellin-responsiveness |
| TCCC-motif | TCTCCCT | Part of a light responsive element |
| W box | TTGACC | Fungal elicitor responsive element |
